# A nationwide survey on neonatal medical resources in mainland China: current status and future challenges

**DOI:** 10.1186/s12887-019-1780-4

**Published:** 2019-11-13

**Authors:** Qiuping Li, Tao Han, Yanping Zhang, Qian Zhang, Xiangyong Kong, Yonghui Yang, Zhichun Feng

**Affiliations:** 1 Neonatal Intensive Care Unit, Affiliated BaYi Children’s Hospital of the Seventh Medical Center of PLA General Hospital, Beijing, China; 2 National Engineering Laboratory for Birth defects prevention and control of key technology, Beijing, China; 3 Beijing Key Laboratory of Pediatric Organ Failure, Beijing, China

**Keywords:** Neonatal, Intensive care, China, Survey

## Abstract

**Background:**

With the rapid development of economy in recent two decades, neonatology has been progressing quickly in China. However, there is little knowledge about the exact developmental status of neonatal departments in China. The aim of this study was to assess resources available for care of sick newborns in mainland China.

**Methods:**

Questionnaires were sent to the membership of the Chinese Neonatologist Association (CNA) and used to survey the scale, facilities, staff, technologies, transport systems and preterm infants’ outcomes of neonatal departments (NDs) in different areas of China from June 2012 to December 2012.

**Results:**

The result of this survey including a total of 117 questionnaires showed that investigated ND had a mean of 65 (median 47; range 5–450) beds, including 19.59 (median 15, range 0–100) NICU beds. The overall doctor/bed and nurse/bed ratio was 1:3.84 and 1:1.43, respectively. Lack of medical equipment was one of the main problems in most NDs surveyed, and only 26 NDs (22.2%) had more than one neonatal incubator per bed. Only 70.1, 30.6, 30.8 and 4.3% NDs carried out high-frequency ventilation, hypothermia, nitric oxide inhalation, and ECMO respectively. The capacity to provide advanced therapies increased with the size of the NDs (*P* < .01). A total of 81 NDs (69.2%) carried out neonatal transport, but only 70 NDs (86.4%) were equipped with transport incubators, 36 NDs (44.4%) had the ability of performing intrauterine transport of the preterm infants, and 3 NDs (3.7%) had the ability of performing air transport. The survival rate of extremely preterm infants (Gestational age less than 28w) to discharge home was 47.8% in 2011.

**Conclusion:**

NDs in mainland China are not well distributed and still face many problems, such as staff shortage, inadequate facilities, and imperfect transport. It is urgent to set up a classification of neonatal care to enhance the utilization rate of medical resources and improve the prognosis of critically ill infants.

## Background

China is the most populous country in the world. According to the 6th National Population Census, the total population of China was 1.34 billion in 2010 [1]. Despite the population and family planning programs, China remains the country with the world’s second largest number of births, second only to India [2]. In recent years, the annual births in China are about 16.00 million. It is a difficult task for China to provide good medical care for the large number of newborns every year. In the mid-1980s, tertiary hospitals in developed areas of China began to establish NICU. With the rapid development of economy in recent three decades, neonatology has been progressing rapidly in China. The most remarkable achievement was the decrease in neonatal mortality from 33.1‰ in 1991 to 9‰ in 2011, although it is still higher than that in developed countries such as the United States, Japan and Australia [3, 4]. Despite the rapid development of NDs in China, there is a shortage of data concerning the overall development status due to the lack of effective management systems and data collaboration networks. The goal of this nationwide survey is to gain insights into the developmental status of NDs in mainland China, probe into current problems and explore directions for future development of neonatal intensive care.

## Methods

### Survey methods

This survey was conducted by the Chinese Neonatologist Association (CNA), and approved by the Medical Ethics Committee of the PLA Army General Hospital (Beijing, China). Altogether 150 hospitals endowed with the membership of the CNA from 31 provinces/municipalities/autonomous regions in mainland China were included in this survey, covering the disciplinary scale, facilities, staffing, technical services, transportation systems and intensive care capacities of the NDs from June 2012 to December 2012. This is not a random sampling survey. Most of the hospitals included in this survey are tertiary hospitals with higher level of neonatal department in their provinces. In order to explore their intensive care capacities, all the patients discharged from the above departments surveyed in 2011 were included in this survey. The questionnaires were sent to the members of CNA by e-mail and the collection procedure was supervised by telephone to minimize possible errors in the questionnaires. Each questionnaire was reviewed and checked by the experts of the CNA. Finally, the qualified questionnaires were recorded and statistically analyzed. The participation in this survey was voluntary without any financial interest.

EpiData Software was used to establish the database. The data were input by two neonatologists independently. Data were compared and corrected if inconsistencies were present. Some of the data obtained from the present survey were compared with the data from another nationwide survey that we conducted in 2008 involving 109 hospitals [5]. Two surveys were largely comparable.

### Statistical analysis

SPSS 18.0 software was used for all statistical analyses of the collected data. Univariate and bivariate analyses were conducted to describe the responses obtained regarding the distribution of neonatal critical care facilities, physicians and nursing resources, and technologic capacities. Pearson’s χ^2^ test and the Kruskal-Wallis test were performed for bivariate comparison of proportions and non-parametric continuous data among NICU categories, respectively. A 2-tailed level of .05 was used as the threshold for statistical significance.

## Results

### Questionnaire recovery and the basic situation of the hospitals included in the survey

Of the 150 eligible hospitals, 117 hospitals responded to the survey with a response rate of 78.0%. Except for Tibet and Qinhai, the 117 hospitals covered almost all provinces/municipalities/autonomous regions of mainland China. Descriptive analysis of the geographical, organizational and logistic characteristics of the NDs investigated is given in Table 1.

**Table 1:** Descriptive analysis of the geographical, organizational and logistic characteristics of the neonatal departments investigated

Characteristics	*n*	%
Region		
East China	26	22.2
South China	19	16.2
Central China	10	8.6
North China	31	26.4
Northwest China	10	8.6
Southwest China	10	8.6
Northeast China	11	9.4
Hospital level		
Level 3	109	93.2
Level 2	8	6.8
Hospital type		
General hospital	71	60.7
Children’s hospital	18	15.4
Maternal & Child Care Hospital	28	23.9
Description		
Independent unit	68	58.1
Belongs to pediatric department	49	41.9
Total beds		
1–20	23	19.7
21–50	47	40.1
51–100	27	23.1
> 100	20	17.1
NICU beds		
0	3	2.6
1–5	25	21.4
6–10	26	22.2
11–20	24	20.5
20–50	32	27.3
> 50	7	6.0
Annual admssions in 2011		
< 1000 infants	32	27.4
1001–2000 infants	39	33.3
2001–3000 infants	14	12.0
3001–4000 infants	14	12.0
> 4000 infants	18	15.3

### Staffing

The 117 hospitals had a total of 1985 newborn care doctors with an overall doctor/bed ratio of 1:3.84, and a total of 5314 newborn nurses with an overall nurse/bed ratio of 1:1.43. There were more admissions in NDs with larger numbers of beds; however, the number of admissions per bed was not statistically different between the NDs of different bed size categories (Table 2). Of note, the doctor/bed ratio and the nurse/bed ratio decreased significantly with the number of beds increasing (*P* < 0 .01) (Table 2).

**Table 2:** Organizational characteristics of neonatal critical care services

	Total	NICU size (No. of beds)				*P*
	(*n* = 117)	1–20(*n* = 23)	21–50(*n* = 47)	51–100(*n* = 27)	> 100(*n* = 20)	
Admissions in year 2011*	1520 (983–3131)	453 (373–758)	1256 (1110–1573)	2739 (2012–3393)	4886 (4014–5557)	< 0.01
Admissions per bed*	33.1 (26.2–44.2)	38.4 (24.1–47.4)	34.2 (29.4–44.4)	33.0 (24.7–50.0)	29.4 (22.2–39.6)	0.403
No. of doctors*	15 (10–21)	8 (5–14)	13 (9–15)	17 (14–20)	26 (21–37)	< 0.01
Doctor/bed*	0.29 (0.21–0.45)	0.60 (0.40–1.05)	0.31 (0.26–0.44)	0.23 (0.18–0.28)	0.17 (0.15–0.23)	< 0.01
No. of nurses*	34 (24–54)	18 (12–24)	29 (25–36)	49 (40–62)	91 (70–118)	< 0.01
Nurse/bed*	0.77 (0.58–1.05)	1.20 (0.93–2.33)	0.87 (0.62–1.07)	0.65 (0.50–0.81)	0.58 (0.41–0.74)	< 0.01

Kruskal-Wallis tests were used for comparisons nonparametric continuous data

*Values reported as median and IQR

### Facilities

The mean number of newborn incubators was 0.73 per cot (range 0.13–2.0) in the 117 hospitals studied, including 0.2 double-layer incubator with adjustable humidity, 0.15 radiant warmer, 0.94 infusion pump, 0.15 ventilator (containing high frequent ventilator), 0.11 continuous positive airway pressure (CPAP), and 0.29 phototherapeutic device per cot. Only 26 hospitals (22.2%) had more than one incubator per cot, indicating that equipment insufficiency was still a problem in many hospitals.

### Therapeutic modalities available

All NDs reported a capacity for tracheal intubation, continuous positive airway pressure (CPAP), blood gas analysis, oxygen therapy, phototherapy, hearing screening, and total parenteral nutrition (TPN). Nearly 100% NDs reported a capacity for conventional mechanical ventilation (97.4%) and surfactant administration (95.7%). The other therapeutic modalities available are given in Table 3. The survey showed that the availability of advanced therapies increased with the size of the NDs (*P* < .01).

**Table 3:** Availability of Therapeutic Modalities in neonatal departments Settings

Modality	Total	NICU size (No. of beds)				*P*
	(*n* = 117)	1–20 (*n* = 23)	21–50 (*n* = 47)	51–100 (*n* = 27)	> 100 (*n* = 20)	
HFOV	82 (70.1)	8 (34.8)	31 (66.0)	25 (92.6)	18 (90.0)	< 0.01
Inhaled nitric oxide therapy*	36 (30.8)	0 (0)	7 (14.9)	14 (51.9)	15 (75.0)	< 0.01
PS replacement	112 (95.7)	20 (87.0)	45 (95.7)	27 (100)	20 (100)	0.093
Exsanguination transfusion	100 (85.5)	10 (43.5)	44 (93.6)	26 (96.3)	20 (100)	< 0.01
Umbilical venous catheter	67 (57.3)	5 (21.7)	23 (48.9)	21 (77.8)	18 (90.0)	< 0.01
PICC	92 (78.6)	12 (52.2)	35 (74.5)	26 (96.3)	19 (95.0)	< 0.01
Hypothermia*	36 (30.6)	0 (0)	10 (21.3)	15 (55.6)	12 (60.0)	< 0.01
Fundus screening	91 (77.8)	9 (39.1)	39 (83.0)	23 (85.2)	20 (100)	< 0.01
ROP laser therapy*	26 (22.2)	1 (4.3)	7 (14.9)	8 (29.6)	10 (50.0)	< 0.01
Bedside ultrasound	100 (85.5)	13 (56.3)	42 (89.4)	25 (92.6)	20 (100)	< 0.01
Neonatal surgery*	73 (62.4)	1 (4.3)	32 (68.1)	23 (85.2)	17 (85.0)	< 0.01
Peritoneal dialysis*	33 (28.2)	0 (0)	8 (17.0)	13 (48.1)	12 (60.0)	< 0.01
CRRT*	16 (13.7)	1 (4.3)	3 (6.4)	4 (14.8)	8 (40.0)	< 0.01
ECMO*	5 (4.3)	0 (0)	0 (0)	2 (7.4)	3 (15.0)	0.028
Neonatal transport	81 (69.2)	9 (39.1)	31 (66.0)	21 (77.8)	20 (100)	< 0.01

Pearson’s χ^2^ test was used for bivariate comparison of proportions. * used Fisher’s exact test

### Neonatal transport

A total of 81hospitals (69.2%) were able to perform neonatal transport, among which 74 hospitals (91.4%) were able to provide this service after 2000. Only 70 hospitals (86.4%) were equipped with transport incubators, and three (3.7%) and 36 (44.4%) hospitals could perform air transport and intrauterine transport for preterm infants, respectively. In 2011, the mean number of transports was 469 (range 4–6572; median 243) including 217 preterm infants (range 2–2685; median 97), and the maximum transport radius was 40–1000 km. Air transport was uncommon, and only three hospitals had the ability to provide air transport service, including one hospital using the helicopter to transport neonates, and the other two hospitals using civil airliners, totaling four times in 2011. The availability of neonatal transportation increased with the size of the NICU (*P* < .01) (Table 3). All the NDs with more than 100 beds could provide neonatal transport.

### Outcomes of preterm infants

**Fig. 1:**
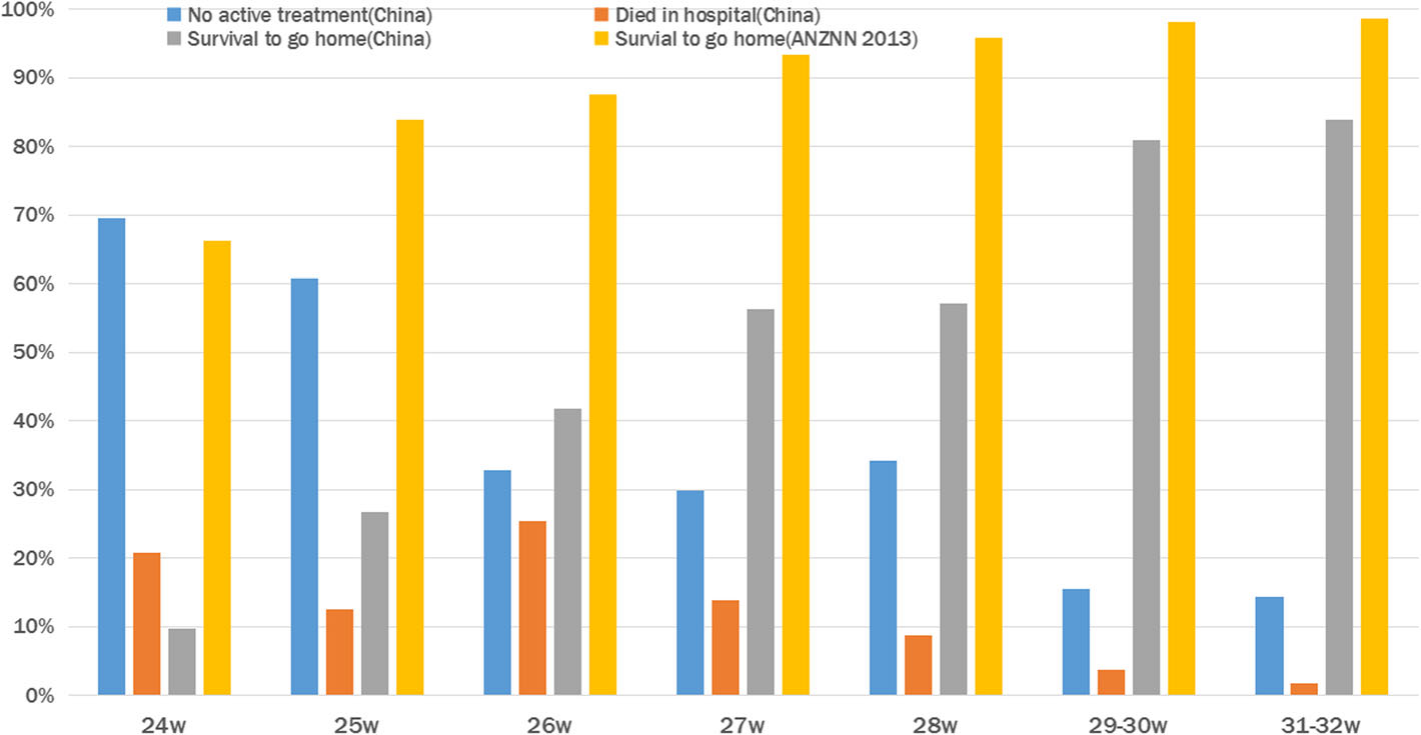
Survival to discharge home by gestational age at birth in 2011

**Fig. 2:**
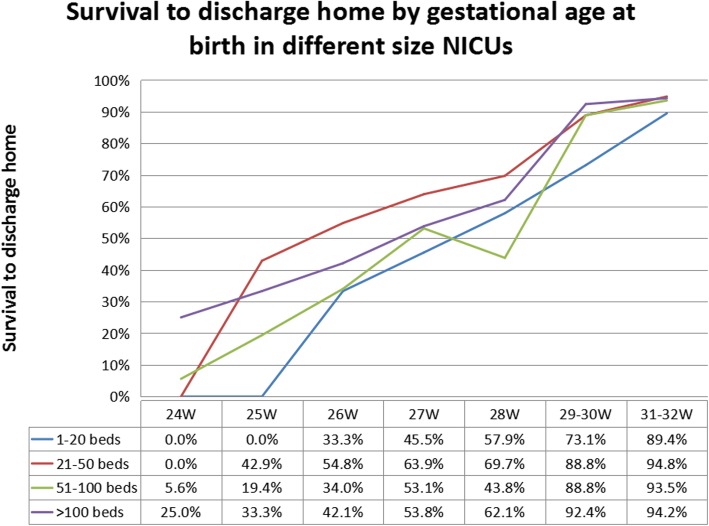
The survival rates of the preterm infants with different gestational ages below 32 w in different size neonatal departments in 2011

In 2011, a total of 250,483 newborns were admitted, with a mean of 2159 per hospital (range 120–8710), including 82,535 (33.0%) preterm infants, and 1841 (0.73%) hospital infant deaths. As some hospitals did not provide detailed newborn data at different gestational ages and birth weights, we only analyzed the admission records of 88 hospitals with complete data. A total of 192,980 neonates were admitted in these 88 hospitals, of whom 62,130(32.2%) were preterm infants, and 577 were extremely preterm infants, accounting for 0.3% of the total admitted infants and 0.9% of the admitted preterm infants. The overall survival rate of the extremely preterm infants was 47.8% in 2011. The survival rates of the preterm infants with different gestational ages below 32 weeks relative to ANZNN data [6] are shown in Fig. 1. The survival rates of the preterm infants with different gestational ages below 32 weeks in different size NDs are shown in Fig. 2.

### Changes in ND development

**Fig. 3:**
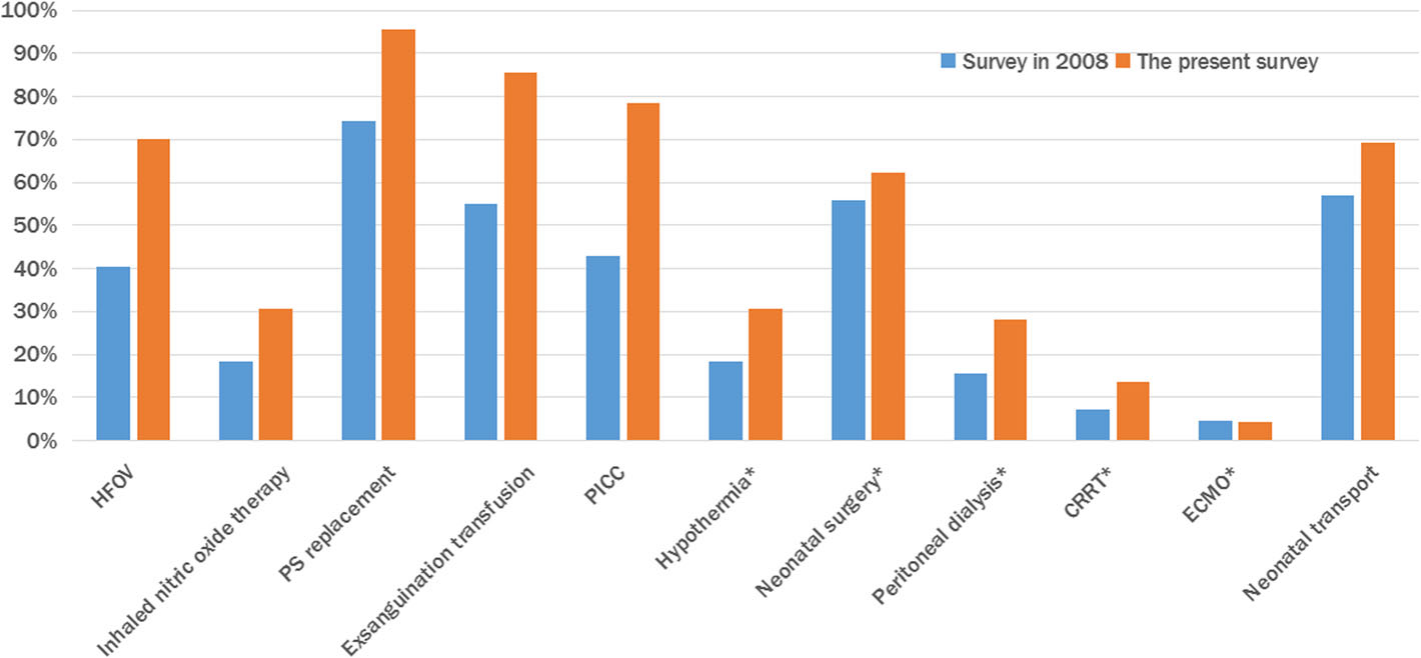
Therapeutic modalities available in 2008 and 2012

Table 4 shows a comparison between the present survey and the 2008 survey in terms of the bed size, number of admissions, doctor/bed ratio, nurse/bed ratio and survival rate of extremely preterm infants. The mean number of beds in the present study was 65 (range 5–450; median 47) vs. 36 (median 30, range 6–300) in the 2008 survey. There was also a significant increase in the mean number of admissions from 1276 per year in the present study to 2159 per year in the 2008 survey. The comparative results of therapeutic modalities available in the two surveys are shown in Fig. 3. Except for ECMO, most technologies including high frequency ventilation, iNO, UVC, hypothermia, peritoneal dialysis, and CRRT in 2012 were more widely available in 2012 than in 2008.

**Table 4:** Comparison between the present survey and the survey conducted in 2008

Item	Survey in 2008 (Total 109 hospitals)	The present survey (Total 117 hospitals)
Mean newborn beds	36(median 30, range 6–300,)	65 (median 47; range 5–450)
Doctor/bed ratio	1:3.24	1:3.84
Nurse/bed ratio	1:1.43	1:1.43
Mean annual admission	1276	2159
Survival rate of extremely premature infants	41.3%	47.8%

## Discussion

This is the first survey report on the status of neonatal departments in mainland China published in the international arena. The results show a rapid progress in both scale and technological development of NDs in mainland China. However, staff shortage, inadequate facilities and disparate development remain the main problems. We also found a significant difference in the availability and accessibility of advanced therapeutic modalities as a function of the number of NICU beds, with the larger NDs (> 100 beds) having such technology available more often. The smallest units (1–20 beds) had the lowest availability and accessibility of advanced technology but had higher ratios of nurses and doctors to beds. Interestingly, it seems that the moderate-size NDs (21–50 beds) have the optimal survival rate of extremely preterm infants as compared with the large-size (> 51 beds) and small-size (1–21 beds) ones.

China began establishing NDs only in some tertiary hospitals in Beijing, Shanghai, Guangzhou and other large cities in the 1980s because of limited economic conditions at that time. Given the small scale of beds, lack of well-trained neonatal doctors and nurses, lack of facilities and equipment, and limited NICU technologic capacity, the survival rate of preterm infants, particularly extremely and very preterm infants, was low [7]. With the rapid development of economy, there is a dramatic increase in the number of NDs in China in recent two decades. It was found in the present survey that more than 50% hospitals have independent NDs with an intermediate number of beds. Compared with the 2008 survey, there is a significant increase in the mean number of newborn beds and annual admissions, indicating that the scale of neonatal treatment institutions in China is developing rapidly. Moreover, the ND size in China is significantly higher than that in some developed countries and India [7–9]. These changes may be attributed to multiple factors. First of all, China has many populous cities, posing great demands on neonatal care. Secondly, NDs in China are mainly set up in tertiary hospitals because of the lack of a classification for neonatal care, thereby resulting in severe centralization of patients. Finally, most families of sick infants tend to seek medical help in hospitals with better equipment and higher technical levels, and few sick infants are transported to lower level hospitals for continuous treatment after their conditions become stable, partly because of the lack of a perfect two-way transport system. Therefore, a large number of sick infants are centralized in high-level hospitals, which results in a shortage of staff, inadequate facilities, and many other issues to high-level hospitals.

In this study, we found that staff shortage remains the main problem in neonatal care in mainland China, especially in large-scale NDs. The overall doctor/bed and nurse/bed ratio is 1:3.84 and 1:1.43, respectively. Compared with the 2008 survey, although the nurse/bed ratio has not changed significantly, the doctor/bed ratio in this survey decreased from 1:3.24 to 1:3.84, indicating that the shortage of doctors in NDs is getting worse. The more beds for newborns in the departments, the more serious the shortage of doctors and nurses. The median doctor/bed and nurse/bed ratio is only 0.17 and 0.58 in the largest neonatal departments (> 100 beds), respectively. Several factors may contribute to the serious shortage of NICU staff in mainland China. Firstly, many NICU doctors and nurses are lost because of heavy workload, low salary, and lack of prestige in NICU care. Moreover, charges for neonatal care are relatively low and the government investment is insufficient. Hence, hospitals cannot afford the annual increases in manpower costs. In the end, the awkward doctor-patient relationship in mainland China makes the situation of NICU staff shortage even worse. Numerous studies have demonstrated that the mortality rate of newborns in different levels of NICUs is related to work load. A British study [10] showed that the mortality rate of a NICU operating at full capacity is 50% higher than that of a same-level NICU operating below capacity. Another British study [11] showed that the number of doctors and nurses is closely related to the mortality rate of preterm infants or very low birth weight newborns. Meanwhile, understaffing may heighten the risks of less attentive care and also increases risks of hospital-acquired infection [12]. .Therefore, China urgently needs to improve staffing shortages in NDs as soon as possible.

The results of the survey show that ND facilities in mainland China are far from sufficiency. There was only 0.73 incubator per bed, and only 0.2 double-layer incubator with adjustable humidity, 0.94 infusion pump, 0.15 respirator, 0.11 CPAP, and 0.29 phototherapy device per bed. Technically, except ECMO, other technologies including high-frequency ventilation, iNO, UVC, hypothermia, invasive blood pressure monitoring, peritoneal dialysis, and CRRT are more popular than those in the 2008 survey. Nevertheless, CRRT, ECMO, and other advanced life support technologies are not widely performed. These advanced life support technologies are only performed in individual NDs of hospitals located in several large-scale cities, including Beijing, Shanghai, and Guangzhou. Hence, training and expanding access to medical technology are important tasks in the future. It is interesting to note greater availability of specific advanced technology and therapeutic modalities in NDs with greater numbers of beds. This is similar to the results of the survey conducted in PICUs in the United States [13].

Many studies [14, 15] have shown that newborn transport networks can significantly reduce the mortality rate of severely premature newborns. China’s newborn transport networks have been developing since 2000, with the mean annual transports of 469 per hospital in 2011, when the proportion of hospitals that could perform newborn transport was significantly higher than in 2008. However, transport facilities are still not perfect, with only 86.4% hospitals equipped with transport incubators. One-way land transport remains the main means of transport in China at present, with a transport distance about 800 km. A study [16] pointed out that long-distance land transport may aggravate the neonate’s clinical condition. The high risk during land transport urges the establishment of an air transport system. However, our survey showed that air transport in China is still in the infantile stage. Only three hospitals (3.7%) can perform air transport, among which two use civil airliners. Air transport can effectively shorten the transport time, but it also increases the cost of transport [17]. China still has a long way to go before a nationwide air transport system can be established. A study [18] showed that intrauterine transport could increase the survival rate of preterm infants. According to our survey, 36hospitals (44.4%) performed intrauterine transport for preterm infants, indicating a great progress in this aspect.

In the present study, the survival rate of extremely preterm infants in mainland China was 47.8% vs. 41.3% in 2008, but still obviously lower than the data from the Canadian Neonatal Network (CNN), Australian and New Zealand Neonatal Network (ANZNN) or National Institute of Child Health and Human Development (NICHD) [6, 19, 20]. This result is similar to the results of another multi-center study conducted in mainland China in 2011, showing that the survival rate of extremely low birth weight infants (ELBWI) was 50% [21]. In addition, only 577 extremely preterm infants were admitted into 88 hospitals in 2011, only accounting for 0.3 and 0.9% of the number and admission rates for preterm infants, respectively. These values are obviously lower than the relative number of births in each category in the general population (about 5% of premature babies) [22], indicating that many extremely preterm infants failed to reach the NICU for intensive care. A multicenter retrospective survey conducted in Guangdong Province of China in 2014, which included 888 cases of extremely preterm infants and ELBWI, showed that 385 cases (43.4%) were discharged without permission or discharged ahead of schedule [23]. Concerns about adverse outcomes and costs may be the main reason for the negative treatment attitude towards extremely preterm infants.

Although this survey was carried out mainly in tertiary hospitals with a relatively large scale, we still found many challenges ahead despite the rapid development of neonatology in mainland China. First of all, the lack of an effective NICU classification management system is an important factor contributing to inappropriate treatment of neonatal patients. Many newborns with mild illnesses occupied valuable intensive care resources. Therefore, health management authorities need to make efforts to establish a NICU classification management system as soon as possible for the sake of utilizing the existing limited medical resources more efficiently. Secondly, a regional, efficient and orderly two-way neonatal transport network needs to be established to solve the transport problem of sick infants between the NICUs of different levels. This can enable critically ill infants to obtain high-level intensive care in time and return to lower-level hospitals for further recover after their condition is stable. Thirdly, the government and hospitals need to give more resources to NDs to improve the facilities and treatment techniques. It is particularly important to improve the remuneration of newborn employees, improve their practice environment, and enhance their professional dignity and identity in order to solve the problem of shortage of newborn care staff. Obviously, there is still a long way to go in China’s newborn care.

## Conclusion

This survey showed that China has made rapid progress in the development of neonatal departments. However, many problems still exist. The main challenges in the future are to increase medical investment on NICUs, solve the problem of neonatal care staff shortage, and establish a classification of regionalized neonatal care and a improve two-way regional neonatal transport network.

The present study has some limitations. The samples were neither inclusive nor randomly selected. Most of these samples were selected from third-level hospitals, some from second-level hospitals, and none from first-level hospitals, knowing that more than 50% of the population in China lives in rural, mountainous and/or remote regions, where most hospitals are at the second or first level. This may result in bias in our sampling. Nevertheless, this survey provides valuable information about the current status of NDs in China, especially those in third-level hospitals.

## Data Availability

Data can be made available on request and following institutional and ethic board approvals for release.

## Abbreviations

CAN: The Chinese Neonatologist Association
ECMO: Extracorporeal membrane oxygenation
ND: Neonatal department
NICU: Neonatal intensive care unit
CPAP: Continuous positive airway pressure
iNO: Inhaled nitric oxide
CRRT: Continuous renal replacement therapy
UVC: Umbilical Vein Catheterization
TPN: Total parenteral nutrition
